# Editorial: Data mining and network-driven analysis: Drug discovery and mechanism exploration by targeting vascular dysfunction

**DOI:** 10.3389/fphar.2022.1099854

**Published:** 2022-12-13

**Authors:** Zhao Dai, Xiaocong Pang, Jiansong Fang

**Affiliations:** ^1^ Science and Technology Innovation Center, Guangzhou University of Chinese Medicine, Guangzhou, China; ^2^ Department of Rheumatology, First Affiliated Hospital of Guangzhou University of Chinese Medicine, Beijing, China; ^3^ Department of Pharmacy, Peking University First Hospital, Beijing, China

**Keywords:** network, vascular dysfunction, drug discovery, data mining, mechanism

Vascular dysfunction refers to the impairment of structural and functional integrity of large vessel (vessel stiffness), microcirculation (microvascular dysfunction), and endothelium (endothelial dysfunction). The integrity of blood vessel structure and function ensures continuous and well-regulated blood supply well match to energy demand, plays a pivotal role in the transport of amino acids, peptides, ions, neurotransmitters, and other signaling molecules, as well as immune surveillance and hemostatic balance. Numerous studies have confirmed that vascular pathology may be a pathogenic factor in the occurrence and progression of dementia, diabetes and cardiovascular diseases (Yang et al., 2022). Clinical-pathological studies support the notion that vascular lesions are detrimental to cognitive function either by directly damaging neurovascular unit maintained in higher integrated functions of cerebral homeostasis or by promoting formation of Alzheimer’s disease (AD) pathology (e g., amyloid plaques) (Fang et al., 2020). Diabetes and cardiovascular diseases share a common vascular related pathophysiological basis. Patients with diabetic retinopathy have more than twice the risk of coronary heart disease (CHD), and also increased risk of fatal disease for congestive heart failure and ischemic stroke (Cheung et al., 2007). Collectively, addressing potential disease interactions associated with vascular dysfunction will greatly deepening our knowledge of the pathogenesis of related diseases. Recent advances in omics data and bioinformatics resources have enabled the rapid development of *in silico* network and data mining-based approaches in complex diseases such as AD (Fang et al., 2021). The Research Topic compiles a series of articles utilizing high-throughput data resources, as well as network pharmacology and computer-based approaches, providing a broad prospect for therapeutic discovery, pathological mechanisms elucidation, and molecular biomarker identification in multiple diseases due to vascular-associated tissue injury. The current Research Topic includes four research articles covering Vascular dementia (VaD), Ischemic stroke (IS), and Pulmonary hypertension (PH).

VaD damages the brain’s arterioles, capillaries, and venules structurally and functionally, leading to cognitive impairment. Nutrients and oxygen are delivered to the brain by blood vessels, which are essential for nerve cells to oxidize and metabolize energy substrates. Therefore, sufficient cerebral blood flow (CBF) is necessary to support neuronal survival and function. The reduction of CBF may contribute to cognitive decline by initiating or amplifying the amyloid cascade. Pathological vascular injury or vascular aging can lead to chronic cerebral hypoperfusion (CCH), which causes various molecular cascades driving downstream structural and functional changes such as blood-brain barrier (BBB) damage, glial activation and neuron damage, which further aggravated cognitive impairment. The current treatment strategies for VaD are performed using acetylcholinesterase inhibitor or N-methyl-d-aspartate (NMDA) receptor antagonist due to a general lack of a reliable molecular biomarker or well defined pathological mechanism. A large number of clinical trials have shown that Traditional Chinese medicine (TCM) therapy can improve the prognosis of debilitating VaD. In this Research Topic, by constructing complex target gene (CTM) miRNA, Liu et al. discovered the potential molecular mechanism of total flavonoids from *Dracocephalum moldavica L* (TFDM) in treating VaD. They found that the neuroprotective effects of TFDM were mainly contributed by kaempferol, apigenin, luteolin, and quercetin. They could act on 43 targets that were mediated by 8 miRNAs. Among them, miR-3184-3p and miR-6875-3p were further validated in oxygen-glucose deprivation (OGD) cell model. This study explored the miRNA expression profile in the process of VaD angiogenesis, uncovered potential pharmacological mechanism of TFDM on VaD, and offered an effective approach to explore the therapeutic mechanisms of TCM. However, given that flavonoids here may be pleotropic compounds that can interfere with bioassays *via* different mechanisms, more in-depth work should be carried out to exclude this possibility (Baell and Nissink, 2018).

IS caused by occlusion of the cerebral artery, where interrupted blood flow to the brain leads to a thrombosis or embolism. Different types of cells undergo different morphological changes during the pathogenesis of IS. In the ischemic core, the cytoplasm and nucleolus of neurons swell and axons disappear. Glial cells transform into an “activated state”, accompanied by the production of pro-inflammatory cytokines and chemokines. Dysregulation of NVU cells plays a vital role in the pathophysiology of IS, which is characterized by disruption of the permeability of BBB and triggering downstream cascades. Reperfusion treatment for IS *via* thrombolysis or thrombectomy agents like alteplase and urokinase are first-line options that reduces disability by restoring blood flow. In addition, reparative therapy of cerebral tissue warrants development for the treatment of IS. However, the lack of treatment options after IS has spurred the search for new stroke therapy. Salvianolic acid for injection (SAFI) serve as a TCM preparation. Previous clinical evidence have suggested a promising prospect for IS since it has no obvious side effects on liver and kidney function. Li et al. utilized network pharmacology and computational prediction as well as experimental verification to systematically analyze the potential mechanism of SAFI on IS. They confirmed 38 common genes shared by SAFI targets and main compound targets as well as genes associated with IS. Among them, PTGS1 and PTGS2 activity were inhibited by SAFI in a dose-dependent manner. SAFI also inhibited LPS-induced prostaglandin E2 production by RAW264.7 macrophages and BV-2 microglia. All the above findings suggest a underlying molecular mechanism of action of SAFI in IS. In addition, Kong et al. used zebrafish to study the protective effect of isopropyl caffeic acid (KYZ) on ischemic diseases, and used network pharmacology and molecular docking to predict angiogenesis through a variety of signaling pathways and predict the target of KYZ toward vascular dysfunction related diseases.

PH is a progressive, fatal vascular disease that is histologically defined by vascular remodeling and clinically characterized by proliferative and apoptosis-resistant cellular phenotypes and adverse pulmonary vascular remodeling, with pathological processes associated with inflammatory cell metabolism. Previous studies have shown that advanced vascular remodeling can be reversed by addressing specific immune processes, and the extent of pulmonary vascular remodeling is closely related to the severity of inflammatory cell infiltration. In physiological states, regulatory T cells (Tregs) take part in suppressing and halting immune responses to prevent autoimmune and tissue damage. Accumulating evidence shows that Tregs are the main cell type of tissue self-tolerance, limiting vascular endothelial damage caused by PH. In this Research Topic, Liu et al. attempted to detect Treg-associated genes (TRGs) in PH patients using transcriptome and Tregs infiltrates data. A gene signature using 25 hub TRGs can better distinguish PH patients from healthy controls. They further verified these predicted genes in lung tissue of PH mouse model. Overall, this study facilitates understanding of the molecular mechanisms associated with Tregs in PH development.

However, several issues should be acknowledged regarding these studies. First, the directly binding targets by TCM or active components have still not validated for corresponding diseases in the current work. In addition, as an essential factor in various disease, endophenotype module for vascular dysfunction can be built to characterize disease pathogenesis for efficacious therapeutic development and biomarker discovery. Third, network-driven analysis tends to highlight highly promiscuous natural products like polyphenols. These compound are usually undruggable or difficult to be drugs. Finally, due to the increasing interdisciplinary technologies that may be used in this field, as well as the complexity of the pathogenesis of vascular dysfunction, more wider scope and in-depth work is still needed.

In summary, the article Research Topic in the Research Topic “Data Mining and Network-driven Analysis: Drug Discovery and Mechanism Exploration by Targeting Vascular Dysfunction” explored the molecular mechanism of drug candidates toward vascular dysfunction related disease and elucidate underlying immune-related mechanisms against diseases due to vascular dysfunction by data mining and network pharmacology.
